# Lipid Peroxidation and Its Role in the Expression of *NLRP1a* and *NLRP3* Genes in Testicular Tissue of Male Rats: A Model of Spinal Cord Injury

**DOI:** 10.22034/ibj.22.3.151

**Published:** 2018-05

**Authors:** Mahshid Bazrafkan, Banafsheh Nikmehr, Abdolhossein Shahverdi, Seyed Reza Hosseini, Fatemeh Hassani, Mahnaz Poorhassan, Tahmineh Mokhtari, Farid Abolhassani, Hamid Choobineh, Cordian Beyer, Gholamreza Hassanzadeh

**Affiliations:** 1 Department of Anatomy, School of Medicine, Tehran University of Medical Sciences, Tehran, Iran; 2 Department of Embryology, Royan Institiute, Tehran, Iran; 3 Department of Urology, School of Medicine, Tehran University of Medical Sciences, Tehran, Iran; 4 Department of Anatomy, School of Medicine, Semnan University of Medical Sciences, Semnan, Iran; 5 Research Center of Nervous System Stem Cells, Department of Anatomy, School of Medicine, Semnan University of Medical Sciences, Semnan, Iran; 6 School of Allied Medical Sciences, Tehran University of Medical Sciences, Tehran, Iran; 7 Institute of Neuroanatomy, RWTH Aachen University, Aachen, Germany

**Keywords:** Infertility, Lipid peroxidation, Testis, Spinal cord injuries

## Abstract

**Background:**

The majority of male patients with spinal cord injury (SCI) suffer from infertility. Nucleotide-binding oligomerization domain-like receptors NOD-like receptors (NLRs) are a kind of receptors that corporate in the inflammasome complex. Recent studies have introduced the inflammasome as the responsible agent for secreting cytokines in semen. Reactive oxygen species (ROS) is one of the elements that trigger inflammasome activation. Genital infections in SCI can lead to ROS generation. We investigated the relation between lipid peroxidation and inflammasome complex activity in testicular tissue of SCI rats.

**Methods:**

Adult male rats (n=20), weighting 200-250 g, were included and divided into four groups: three experimental groups, including SCI1, SCI3, and SCI7, i.e. the rats were subjected to SCI procedure and sacrificed after one, three, and seven days, respectively and a control group. We performed a moderate, midline spinal contusion injury at thoracic level 10. The animals were anesthetized, and testes were collected for measurement of gene expression by real-time PCR. Caudal parts of epididymis were collected for malondialdehyde (MDA) measurement.

**Results:**

No *NLRP1a* mRNA overexpression was seen in the testes of control and SCI groups. After seven days from SCI surgery, *NLRP3* mRNA expression was significantly increased in SCI7 animals (*p* ≤ 0.05). There was a significant difference in MDA level in SCI7 versus control group, as well as SCI1 and SCI3 animals (*p* ≤ 0.05).

**Conclusion:**

*NLRP3* overexpression occurs due to the increased ROS production in testis tissue of SCI rats

## INTRODUCTION

Every year, spinal cord injury (SCI) caused by war and accidents afflicts numerous individuals. The mean frequency of this injury is annually 10,000 persons/year in the United States[1] and approximately 2000 persons/year in Iran[2]. Most commonly, the victims include young men in the prime of their fertility, but only 10% of these patients may procreate without the help of assisted reproductive technologies. Therefore, the issue of fertility in men with SCI constitutes a major source of psychological tension and stress in these patients[3-7]. The most important causes of infertility secondary to cord injury include disorders of erection, ejaculation, and semen abnormalities[8]. Although the electroejaculator technology may overcome, to a great extent, the erectile and ejaculatory dysfunctions, semen samples collected in this way are of poor quality. Leukocytospermia, teratozoospermia, elevated number of immature sperms, poor motility, necrospermia, increased sperm DNA fragility, and high semen viscosity are common problems in these samples[9]. Moreover, DNA damage is one of the main effects of SCI on spermatozoa[10].

Different etiologies have been proposed to account for the poor quality of semen following SCI, including changes in urination pattern[11], scrotal hyper-thermia[12], hormonal disorders[13], and changes in sperm transportation and retention due to stasis[14,15]. None of these, however, have been proven conclusively[7,9].

Recent studies have indicated that increase in inflammatory cytokines, such as IL-1β and IL-18, in seminal plasma of SCI patients is toxic to sperm and affects semen quality[16]. The association between increased inflammatory cytokines in semen and poor sperm quality implicates the innate immune system and inflammasome complex as important factors contributing to infertility in SCI[17,18].

The innate immune system initiates the defense response and activates the adaptive immune system. A key element of the innate immune system is responding through NOD-like receptors (NLRs), which are able to identify noxious stimuli within cytoplasm[19-21]. NLRs contribute to the formation of a cytoplasmic complex, known as inflammasome, which is composed of a multiprotein complex to activate caspase 1 and subsequently, IL-1β and IL-18. Caspase 1 is a cysteine protease that initiates and performs pyroptosis−a form of programmed cell death[22,23]. *NLRP1a* was the first gene to be identified in association with this complex; nevertheless, little is known about its activators. Its best known activation element is lethal antrax toxin[23]. *NLRP3* is another well-known gene of the inflammasome complex that is activated in various diseases[24-27]. It has recently been recognized as a factor contributing to testicular tissue disruption in the ischemia reperfusion model[28]. Oxidative stress in cells is a major activator of the inflammasome complex[29]. A common event following SCI is testicular and epididymal inflammation[30], which paves the way for infiltration of leukocytes or their mediators[20,31]. Leukocytes constitute the most important source of reactive oxygen species (ROS) production, in order to control infection via these biochemically active molecules[32]. On the other hand, production of a large number of ROS creates oxidative stress inside the cells, contributes to inflammasome complex activation[33].

So far, two studies have been conducted on the presence of inflammasome components in the ejaculatory fluid; their results indicated that inhibiting these components in patients with SCI improves sperm motility[17,18]. Considering the fact that inside the testes, spermatogonial stem cells are exposed to pathogens as well as to inflammatory cells and their mediators, testicular infection, and inflammation will pose a greater risk to the reproductive function, as compared to afflictions of the accessory glands[20,31]. Previous studies have paid less attention to the inflammasome complex in the testis and its activators following SCI. Regarding the crucial role of inflammation and oxidative stress in disrupting testicular function, this study aimed to investigate the expression of inflammasome complex genes in testicular tissue and its association with oxidative stress at three time points during the acute phase of injury. The findings may contribute to development of new treatment interventions for patients with SCI.

## MATERIALS AND METHODS

### Animal and housing conditions

In this study, we used 20 male adult Wistar rats weighing 200-250 g. In accordance with the regulations of the ethics committee of Tehran University of Medical Sciences (Iran), the animals were housed in groups of two in special cages with food and water provided *ad libitum*. Their environment followed standard 12-hour dark and 12-hour light cycles, without sound pollution, regularly monitored and preserved at 22 ± 2 °C. Starting on the day after surgery, the rats were administered enrofloxacin (1 mg/kg) for 10 days and dextrose serum for 7 days subcutaneously. After surgery, the bladder was emptied on a daily basis or twice daily, if necessary. Over the period of seven days, cage beddings were renewed daily, and the animals were assessed for bed sores every day.

### Laminectomy and surgery for induction of SCI

**Fig. 1:**
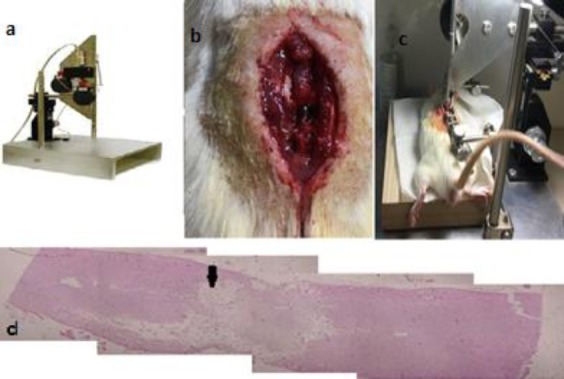
Tools needed to create a spinal cord injury model and model confirmation. (a) NYU MASCIS (New York University, Multicenter Animal Spinal Cord Injury Study) apparatus; (b) cord bruising following the lesion; (c) the animal’s tail reflex immediately after the impact of rod on spinal cord; (d) longitudinal section of the spinal cord depicting glial scar and cavity at the level of T10 and confirming the contusion/compression model.

The entire process of laminectomy was performed under sterile conditions. In order to develop a model of spinal cord contusion, the animal was first anesthetized with a mixture of ketamine (80 mg/kg) and xylazine (10 mg/kg). After confirming anesthesia, the animal’s back was thoroughly shaved. Since our lab model of SCI was set at the level of T10, this vertebra was identified by palpation[34-36]. Then using a sharp scalpel, a 3-cm incision was made on the skin; connective tissue and muscles on both sides of the vertebral column were pushed aside and held in place with retractors. The lamina of T10 vertebra was removed to expose a surface proportionate to the rod of the impactor device. Subsequently, the animal was transported to the NYU MASCIS (New York University Multicenter Animal Spinal Cord Injury Study) impactor. After fixing vertebrae 9 and 11 using spinous process clamps and after confirming the alignment of the rod over exposed spinal cord, the 10-g rod was dropped from a height of 25 mm. After 1 to 3 minutes, cord contusion was prepared (Fig. 1). Once the rod is dropped, three events may confirm the development of contusion: first, bruising of the spinal without disruption of the dura at the impact site (Fig. 1), second, wagging of tail intensively, and third, bruising and flaccidity of the animal’s both plants. Finally, the animal was sutured: initially, the connective tissue was closed using 3-0 absorbable filament, and then skin was repaired with 4-0 non-absorbable filament. The animal was placed on a warm plate (37 °C) to regain consciousness. After surgery, enrofloxacin and normal saline (5 mL) were administered subcutaneously.

### Categorization

The rats were randomly categorized into four groups (five animals per group) as follows: SCI1, sacrificed on the day after surgery; SCI3, sacrificed three days after surgery; SCI7, sacrificed seven days after surgery; the control group which did not undergo surgery

### Behavioral test

Twenty-four hours after the surgery, the animals were evaluated using the Basso, Beattie, Bresnahan Locomotor Rating Scale (BBB)[37]. Animals with grade 0 BBB results entered the study. In order to confirm the model of spinal injury, slides of T10 with Hematoxylin and Eosin (H&E) staining were prepared from some animals with grade 0 BBB.

### Removing testis and epididymis

Rats were anesthetized with high-dose ketamine/xylazine. A small incision was made on the scrotum, and the epididymis was extracted and preserved in Ham’s F10 (Gibco, USA) solution in an incubator at 37 °C for subsequent studies. Next, the animal’s chest was opened, and perfusion was performed with 1000 cc normal saline containing 1 cc heparin until the testes turned completely white, and the tissue became void of blood cells. The testes were then extracted.

### RNA extraction and cDNA synthesis

Total RNA was extracted from testicular fragments using TRIzol solution as described by manufacturer (Sigma-Aldrich, St Louis, MO, USA). Qualitative evaluation of the extracted RNA was performed using a NanoDrop instrument[38]. cDNA synthesis was carried out by SCRIPT cDNA synthesis kit (prime synthesis kit, TAKARA, Japan). All the steps were performed in a thermal cycler (Eppendorf, Germany), and the final product was kept at -20 °C.

### Primer preparation

In this study, we used the β-actin housekeeping gene, as the internal control. Initially, the primers were designed using Perl Primer V1.1.21. The NCBI/Primer-BLAST was used to confirm their specificity for the template (Table 1)

**Table 1:** Primers sequences

Gene	Primer sequence (5’→3’)	Accession number
*NLRP1a*	Forward: CTGCCGACTAAAGACCTTGTG	NM_001145755.2
	Reverse: GTCTAGTTCAGCCAACCTTGAG	
*NLRP3*	Forward: AGCCAACCATCTCTTCTTCC	NM_001309432.1
	Reverse: AAGTCATGCTGTAGCCAACC	

### Real-time PCR

Real-time PCR was used for quantitative assessment of inflammasome genes. The reaction was performed using SYBR Green and at a total volume of 20 µL in each well. The reaction mixture was prepared by mixing 5 µl SYBER Green 5, 1 µl forward primer, 1 µl reverse primer, 2 µl cDNA, and 11 µl water. Then the thermal protocol was implemented as follows: first denaturation, 10 minutes at 95 °C, primer annealing and extension for each cycle, as well as fluorescence reading at the end of each cycle. Data were analyzed with the 7000 Applied Biosystem SDS software (version 201) using CTΔΔ method.

### Quantifying lipid peroxidation

The concentration of epididymal sperm malondialdehyde (MDA) was measured spectro-photometrically using the thiobarbituric acid (TBA) method. Initially, the epididymis tail was placed in 0.5 mL previously warmed Ham’s F10 (Gibco, USA) solution containing FBS. The epididymis tail was perforated in several spots to allow sperms to enter the solution. Using computer-assisted sperm analysis[39], the number of sperms in each sample was determined. Then a volume was acquired to achieve the equal numbers of sperms in all samples. Also Ham’s F10 solution was added to the acquired sperm specimen to obtain a final volume of 1 mL. Subsequently, this solution was place in boiling water (95 °C) bath for 15 minutes with 2 mL TBA mixture (1 : 2) containing 15% chloroacetoacetic acid, TBA, and 0.25 normal hydrochloric acid; all ingredients were procured from Sigma Alderich, Germany. Afterwards, the solution was cooled with running water. Eventually, after centrifugation at 1789 ×g for 20 minutes, the supernatant was removed and its light absorption at 532 nm was read. MDA concentration was calculated using an extinction coefficient of 1.56 × 10^5^ mol^-1^.L.cm^-1^ for the MDA-TBA complex and expressed in μM[38].

### Light microscopy

For routine histological preparations, the tissue samples were fixed and dehydrated in ascending grades of ethanol, cleared in xylene and embedded in paraffin wax. The sections (5 µm) were cut on a microtome and stained with Harris H&E[40].

### Statistical analysis

All statistical analyses were performed using SPSS software version 22. The results were expressed as means ± SEM. Parametric variables were analyzed using one-way analysis of variance (ANOVA), *t*-test, and Tukey’s test. Non-parametric variables were analyzed using Kruskall-Wallis test and Dunn’s multiple comparisons for post test. *P* values <0.05 were considered statistically significant.

## RESULTS

### Expression of NLRP1a and NLRP3

**Fig. 2:**
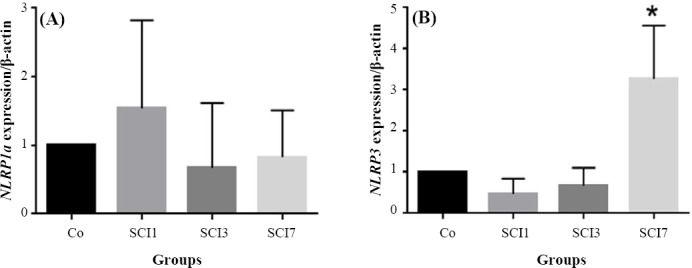
Evaluating the expression of *NRPL1a* and *NRPL3* in rat testes after induction of SCI. Co, control group; SCI1, group sacrificed on day one after induction of SCI; SCI3, group sacrificed on day three after induction of SCI; SCI7, group sacrificed on day seven after induction of SCI. ^*^ *p* < 0.05 compared to the control group

In this study, we assessed the expression of *NLRP1a* and *NLRP3* genes in testis tissue. Our results indicated no significant difference in the expression of *NLRP1a* between the days studied (Fig. 2A). Nevertheless, the expression of *NLRP3* was significantly different between the SCI7 group and the other groups (*p* < 0.05; Fig. 2B).

### MDA quantification

**Fig. 3:**
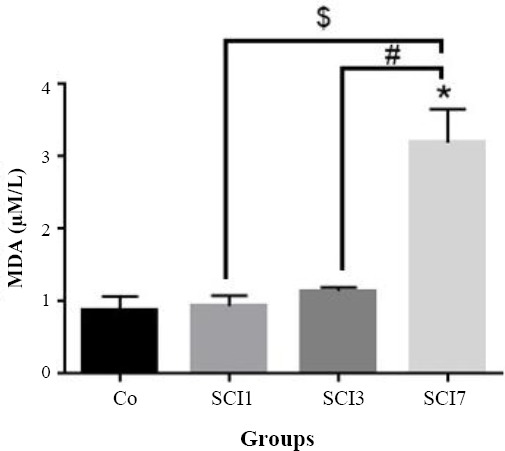
Quantification of malondialdehyde (MDA) in rat testes after induction of SCI. Co, control group; SCI1, group sacrificed on day one after induction of SCI; SCI3, group sacrificed on day three after induction of SCI; SCI7, group sacrificed on day seven after induction of SCI. ^*^ *p* < 0.05 compared to the control group; ^#^ *p* < 0.05 compared to SCI group, ^$^ *p* < 0.05 compared to SCI7 group

**Fig. 4:**
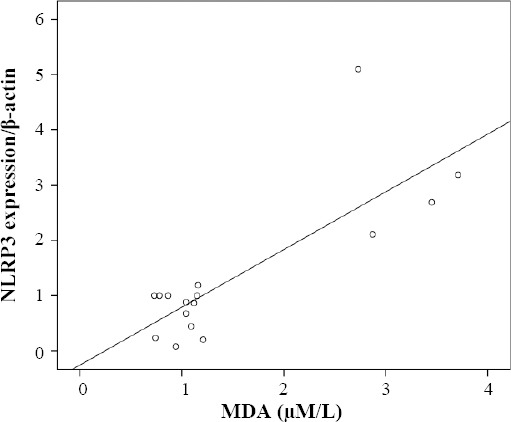
The correlation between malondialdehyde (MDA) and *NLRP3* expression in rat testes after induction of SCI (*p* = 0.0001, r = 0.803). By increasing the MDA, the expression of *NLRP3* was enhanced.

In this study, testicular tissue MDA was assessed and, based on the findings, a significant difference in MDA concentration between the group SCI7 and the others was observed (*p* < 0.05; Fig. 3). There was a significant correlation between MDA and *NLRP3* expression in rat testes after the induction of SCI (*p* = 0.0001, r = 0.803, Fig. 4).

### H&E staining

**Fig. 5:**
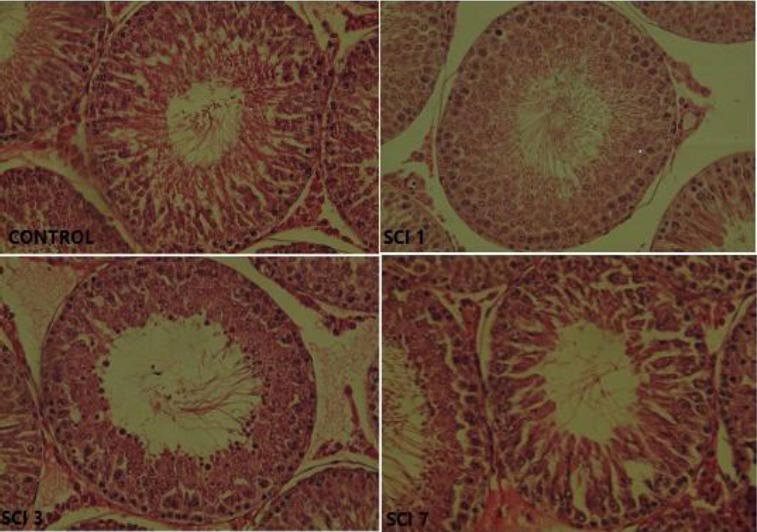
H&E staining from testis transverse sections (400× magnification). Control, control group; SCI1, group sacrificed on day one after induction of SCI; SCI3, group sacrificed on day three after induction of SCI; SCI7, group sacrificed on day seven after induction of SCI

Based on Figure 5, there was no significant difference in spermatogenesis between the control as well as SCI1 and SCI7 groups. A moderate regression was seen in spermatogenesis in the testicular tissue of SCI7 rats.

## DISCUSSION

In this study, we investigated lipid peroxidation and its role in the expression of inflammasomes complex (*NLRP3* and *NRPL1a* genes) in male rat testicular tissue following SCI. Our results showed that seven days after SCI, the MDA concentration and *NLRP3* expression level were significantly increased in parallel.

Infertility is one of the complications of SCI, caused by erection and ejaculation disorders as well as disorders of the semen[4] such as asthenospermia, necrospermia, and DNA fragmentation, which occur extensively since the first days after injury[7,32]. Different etiologies have been proposed for these disorders, including scrotum hyperthermia, hormonal deficits, disorders of the hypothalamus-pituitary axis, and disorders of the blood-testis barrier; however, none of which have been proven unequivocally.

In 1990, Hirsch *et al*.[41] was the first one who mentioned the crucial role of the immune system in poor sperm motility. He reported that the role of the immune system must be addressed when treating infertility in these patients. The findings of a study in 1996 indicated that following SCI, the high level of inflammatory cytokines in semen makes it toxic for sperms[42]. Ever since, activation of the immune system has been accepted as a major contributor to infertility after SCI.

In 2011, Dulin *et al*.[43] reported that 72 hours after SCI in rats, the integrity of the blood-testis barrier is disrupted, with a considerable increase in the apoptosis of germinal cells and IL-1β. They attributed these findings to intense immune response in the testes, although they could not provide an exact mechanism. Two recent studies have addressed an important component of the immune system, namely the inflammasome complex, as a contributing factor to infertility following SCI. In the first study, the presence of IL-1β and IL-18 in semen from patients with SCI was attributed to the activation of the inflammasome complex. In the subsequent study, some components of the complex were identified in sperms, and administering antibodies against them was reported to improve sperm motility[17,18]. These two studies failed to exactly demonstrate whether the testes or accessory glands are the source of the inflammasome complex in semen. Most investigations attribute seminal leukocytes to the prostate and seminal vesicles[44]. Therefore, it may be presumable that the abundant leukocytes present in semen after SCI might secrete the components of inflammasome into semen. The current study aimed to evaluate the expression of two genes, which are the upstream of the inflammasome complex, in testes where the sensitive spermatogonia begin the process of spermatogenesis. The findings will reveal the role of testes alone in expression of inflammasome genes and infertility following SCI.

Inflammation of the testis and epididymis is a common problem of the reproductive system after spinal cord lesions[32]. This problem will give rise to the condition in which immune cells infiltrate the testis and epididymis to produce considerable oxidative stress with high level of oxygen consumption[20,31]. In our study, we observed high levels of MDA production in epididymal sperm 72 hours and one week after

the SCI, indicating lipid peroxidation. The findings demonstrate that seven days after the cord injury, simultaneously with elevated oxidative stress, the expression of *NLRP3* was significantly increased. As mentioned in a previous study, reactive ROSs constitute an activator of the inflammasome complex[45], which is corroborated by our findings. In a study conducted by Iremashvili *et al*.[7] in 2012, white blood cells were incriminated as the source of IL-1β in semen. On the other hand, our findings suggest that following SCI, testicular cells with activated inflammasome complex may also contribute to the elevated seminal level of IL-1β.

Numerous studies have reported extensive apoptosis of sperm and testicular cells after SCI[46,47]. Talebi *et al*.[10] investigated the integrity of nuclear DNA in epididymal spermatozoa following chronic SCI and showed a disruption in sperm parameters following chronic SCI in rats, including DNA integrity of sperms. It is known that the high level of unsaturated fatty acids in the cell membrane of sperms renders them susceptible to lipid peroxidation and production of MDA[4]. Combination of MDA with DNA[45] may trigger apoptosis or pyroptosis resulting from inflammasome activation, which can manifest as TUNEL-positive cells or necrospermia. As a corroboration for this assumption, the MDA produced by monosodium urate has been shown to result in DNA alterations in dendritic cells and induction of *NLRP3* activation[45]. As mentioned above, activation of the inflammasome complex eventually leads to a form of programmed cell death, known as pyroptosis. Despite the difference in cell types, the findings are consistent with our results regarding the mechanism of *NLRP3* activation. In 2015, a study by Minutoli[28] on a testicular ischemia/reperfusion model showed that 24 and 72 hours after surgery, the expression of downstream genes of the inflammasome complex was significantly increased in testes of mice with suppressed *NLRP3* gene compared to the normal model. This is the only study to explicitly address the inflammasome complex in testis and reports the high level of ROS as the major factor in the signaling cascade, leading to the disruption of spermatogenesis and activation of the inflammasome complex. Based on the mentioned study, a significant increase in expression of downstream genes of inflammasome was observed after 24 hours of the injury, whereas our findings did not indicate any increase during the first 72 hours. This discrepancy may reflect the difference in methodologies. While the study by Minutoli[28] inflicted a direct change on the testes to develop a model of ischemia, we chose to create the lesion at the level of T10, which does not affect testicular innervation and perfusion directly. Therefore, we attempted to develop a model to study the impact of SCI on the testes in a more precise manner. In one of the first studies addressing this issue, Huang *et al*.[48] reported apparent abnormalities in spermatogenesis of rats within one week after SCI. In another studies, the qualitative and quantitative impairment of spermato-genesis in rats occured during the acute phase of SCI[48,49]. In addition, Choobineh *et al*.[28] observed that the greatest reduction in the amount of testosterone occurs during the first week after SCI. Considering these findings as well as those of Dulin *et al*.[43] mentioned above, we designed this study to address the acute phase during the first week after SCI.

The expression of *NLPR1α* was not increased significantly after SCI. Various reasons may account for this findings; for instance, the oxidative stress may not act as an inducer for this gene. Furthermore, our study only covered the acute phase of injury, and it is possible that increased expression may occur after one week.

Our study appears to be the first to address the expression of inflammasome genes in testes in a model of SCI at three time points during the acute and sub-acute phases. Further studies are required to determine exactly which cells express this gene in the testes. It seems that the concomitant expression of *NLRP3* and increased production of quantifying lipid peroxidation may be considered as a key target for treating infertility in such patients. Creating a balance between oxidants and antioxidants in these patients may minimize the activity of inflammasome and might improve their fertility and the quality of their life.
